# Hypotension and Bradycardia After Brachiocephalic Artery Stenting: A Case Report

**DOI:** 10.7759/cureus.75249

**Published:** 2024-12-06

**Authors:** Masahiro Morishita, Takaaki Yamazaki, Hiroshi Moriwaki, Makoto Senoo, Mikio Nishiya

**Affiliations:** 1 Department of Neurosurgery, Hakodate Neurosurgical Hospital, Hokkaido, JPN

**Keywords:** acute ischemic stroke, brachiocephalic artery stenosis, brachiocephalic artery stenting, bradycardia, evt: endovascular therapy, hypotension, stent placement

## Abstract

Angioplasty and stenting of brachiocephalic artery stenosis can be complicated by ischemic stroke, local hematoma, thromboses, or dissection of access vessels. However, hemodynamic instability has not been reported as a complication of this treatment. We report the case of an 83-year-old man who developed hypotension and bradycardia after brachiocephalic artery stenting. He was admitted to our hospital with a right frontal infarct and severe brachiocephalic artery stenosis with cerebral hypoperfusion and subclavian steal syndrome. We performed brachiocephalic artery stenting with prophylactic atropine administration, during which he was hemodynamically stable. Post procedure, however, he developed hypotension and bradycardia requiring an atropine and dopamine drip. This hemodynamic instability resolved in approximately 12 hours and did not recur. He experienced no complications associated with this hemodynamic instability. This report provides evidence that hypotension and bradycardia can occur after brachiocephalic artery stenting.

## Introduction

Brachiocephalic artery stenosis has been safely treated in recent years with endovascular therapy. Angioplasty and stenting of this lesion can be complicated by ischemic stroke, local hematoma, thromboses, or dissection of access vessels [1]. Although hypotension and bradycardia after carotid artery stenting are well recognized [2,3], such hemodynamic instability has not been reported as a complication of brachiocephalic artery stenting. Moreover, the appropriate management of this complication is not well known. We report a case of brachiocephalic artery stenosis in which hypotension and bradycardia occurred after stent placement, and atropine and vasoconstrictors were effective for perioperative management.

## Case presentation

**Figure 1:**
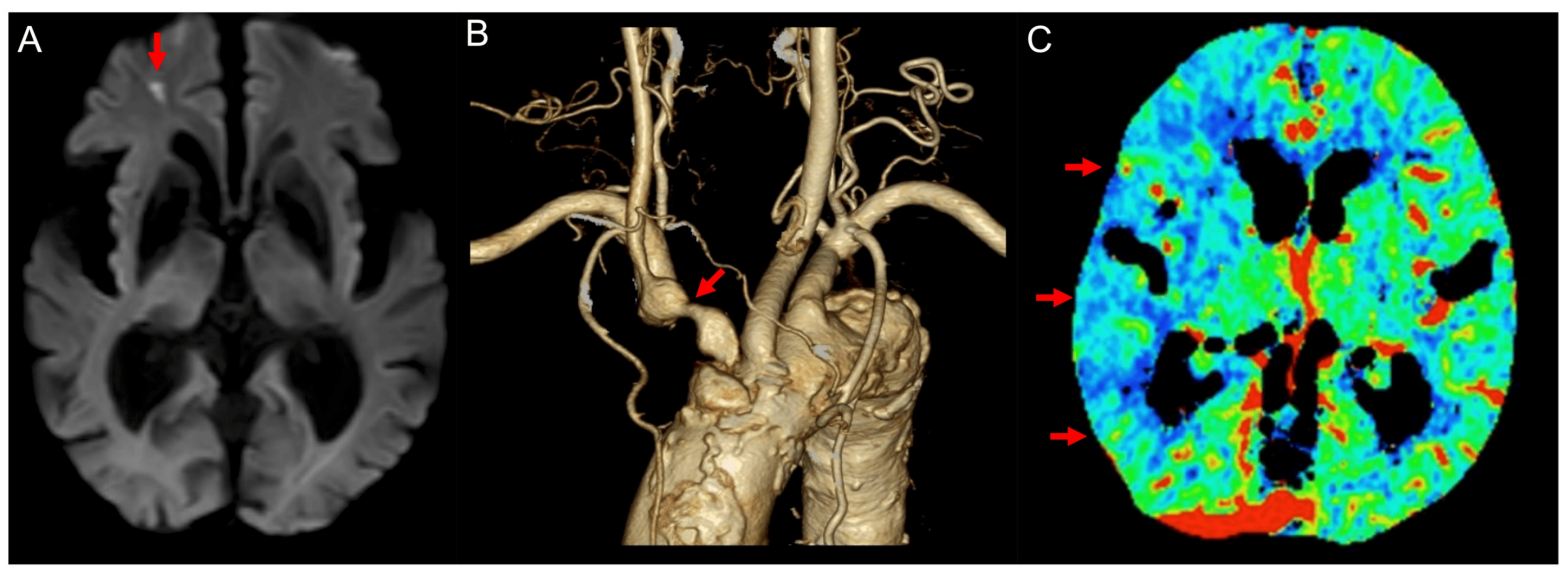
Preoperative images Magnetic resonance imaging on admission showed an infarct in the right frontal lobe (A) (arrow). Computed tomography angiography and perfusion imaging showed severe brachiocephalic artery stenosis (B) (arrow) and decreased cerebral blood flow (C) (arrows).

An 83-year-old man with a history of hypertension and coronary artery disease was admitted to our hospital with an asymptomatic infarct in the right frontal lobe (Figure 1A) in July 2023. He had been treated with a calcium channel blocker and a single antiplatelet agent. Magnetic resonance angiography on admission showed reduced signal intensity of the right internal carotid artery. Severe brachiocephalic artery stenosis and decreased cerebral blood flow were detected on computed tomography angiography and perfusion imaging (Figures 1B, 1C).

**Figure 2:**
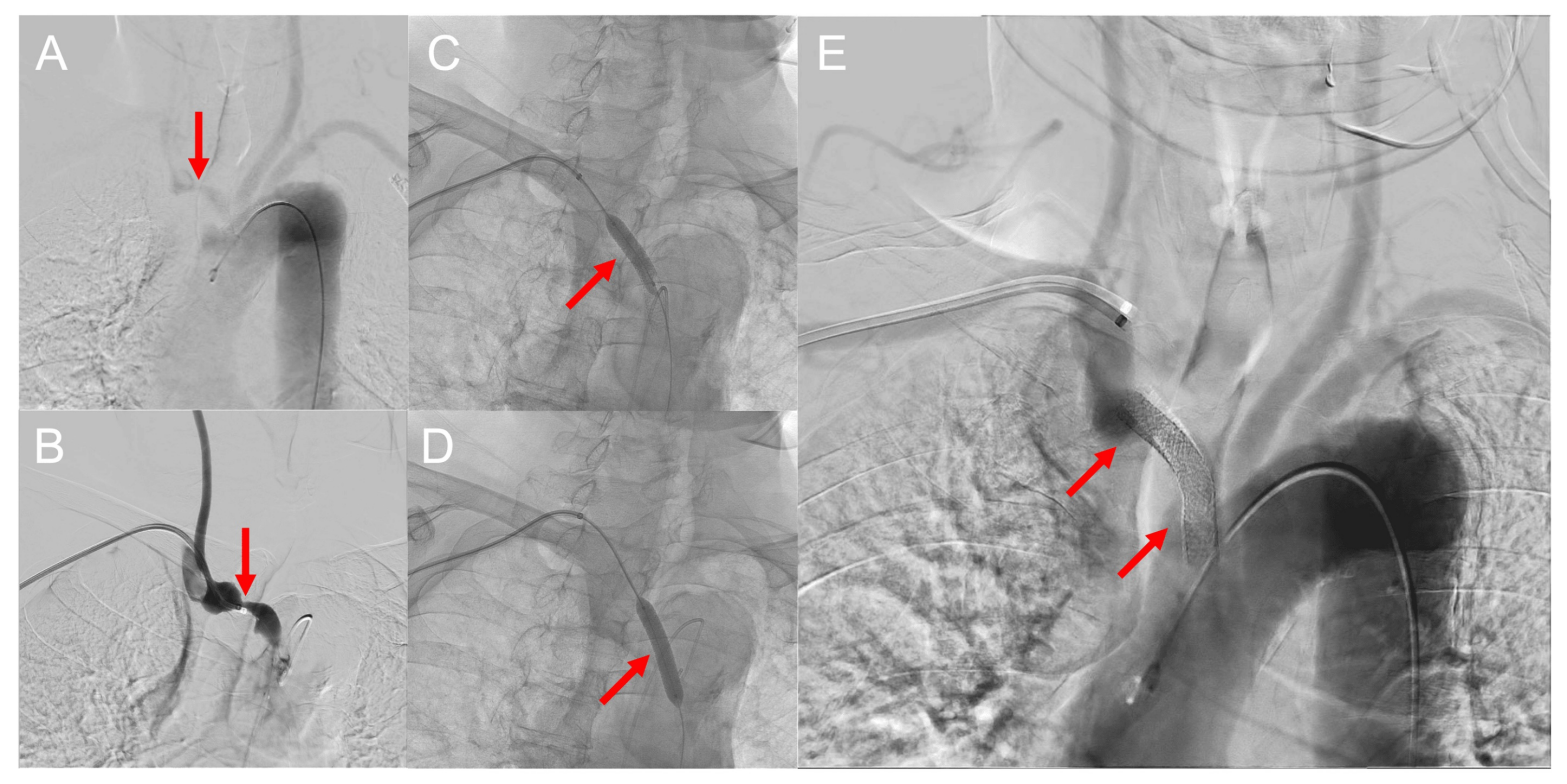
Brachiocephalic artery stenting Aortic arch angiography showed severe stenosis of the brachiocephalic artery (A, B) (arrows). Angiography after placement of two express LD stents (each stent was 8 × 37 mm) showed sufficient dilatation of the brachiocephalic artery (C, D, E) (arrows).

He also had a considerable brachial systolic blood pressure difference of 34 mmHg. We diagnosed him with severe brachiocephalic artery stenosis with cerebral hypoperfusion and subclavian steal syndrome. We started dual antiplatelet therapy and performed stent placement 18 days after admission. Endovascular treatment was performed under local anesthesia. Heparin was administered during the treatment. A 90 cm 6 Fr FUBUKI XF guiding sheath (ASAHI INTECC, Aichi, Japan) was guided to the brachiocephalic artery. We used prophylactic atropine to reduce the risk of hemodynamic instability in advance and placed two balloon-expandable Express LD stents (each stent was 8 × 37 mm, Boston Scientific, Natick, MA, USA) in the brachiocephalic artery. Postprocedural angiography showed sufficient dilatation of the brachiocephalic artery (Figure 2).

He was hemodynamically stable during the procedure but developed dizziness with a sustained low systolic blood pressure < 90 mm Hg and a low pulse rate < 50 beats/minute two hours after the procedure. He did not have any history of bradyarrhythmia or any medication that could cause bradycardia, such as beta blockers. Laboratory data did not show electrolyte imbalance, which could cause bradycardia. We administered an intravenous atropine and dopamine drip. This hemodynamic instability resolved in approximately 12 hours and did not recur. He experienced no complications associated with this hemodynamic instability. Cerebral hypoperfusion on computed tomography perfusion imaging and the brachial systolic blood pressure difference improved to less than 10 mmHg after the procedure. We followed the patient for 12 months and observed no recurrent stroke on magnetic resonance imaging.

## Discussion

There were two important clinical implications based on our findings. First, hypotension and bradycardia can occur after brachiocephalic artery stenting. Second, atropine and vasoconstrictors are effective agents to prevent and treat perioperative hemodynamic instability. These implications are discussed below.

We found that hypotension and bradycardia can occur after brachiocephalic artery stenting. Baroreceptors have been identified in the carotid sinus and the aortic arch in humans [4]. These baroreceptors are stimulated by mechanical deformations of the arterial wall. The signal is propagated along the afferent nerves to the central nervous system and serves as negative feedback on blood pressure and heart rate [5]. Carotid artery stenting is thought to stretch carotid sinus baroreceptors and cause hypotension and bradycardia with an incidence of 5% to 76% [2, 3]. Animal experiments have shown that baroreceptors are also present in the wall of the brachiocephalic artery [6]. Therefore, brachiocephalic artery stenting can also cause hemodynamic instability, as found in our patient. Another presumptive mechanism for the findings, in this case, is that brachiocephalic artery stenting improved blood flow and increased blood pressure in the carotid artery and may have stretched carotid sinus baroreceptors as a result [4]. The third possible mechanism is that baroreceptors in the aortic arch were stimulated by the extra stent protruding into the aortic arch [4]. To the best of our knowledge, there have been no reports on hypotension and bradycardia after brachiocephalic angioplasty. Therefore, the appropriate management of this complication is not well known. This report provides additional evidence that hypotension and bradycardia can occur after brachiocephalic artery stenting.

Our findings also suggest that atropine and vasoconstrictors are effective agents to prevent and treat perioperative hemodynamic instability. These agents are usually administered for hemodynamic instability in carotid artery stenting [7]. Atropine is a parasympatholytic drug that enhances sinus node and atrioventricular conduction and is effective in patients with bradycardia due to heightened parasympathetic tone [8]. In one study of 105 cases of carotid artery angioplasty, 37 of 39 (95%) patients with prophylactic atropine did not experience bradycardia during the procedure, although 26 of 66 (39%) patients without prophylactic atropine experienced hemodynamic instability [9]. In our patient, prophylactic atropine may also have led to hemodynamic stability during the procedure. Moreover, postoperative hemodynamic instability was transient and could be managed with atropine and dopamine. The importance of cardiovascular components in endovascular treatment has been emphasized in a previous study [10]. It has been reported that severe hemodynamic instability associated with carotid artery stenting can result in neurologic sequelae [3, 7]. In our case, prophylactic atropine could have prevented severe hemodynamic instability and subsequent neurologic sequelae associated with brachiocephalic artery stenting. Hemodynamic instability could also be associated with postprocedural complications not only in carotid artery stenting but also in brachiocephalic artery stenting. Future studies are warranted to examine the effects of hemodynamic instability after brachiocephalic artery stenting on patient outcomes. Clinicians should not neglect this cardiovascular complication of brachiocephalic artery stenting. In carotid artery stenting, advanced age, a history of coronary artery disease, and calcification of stenotic lesions are known risk factors for hemodynamic instability [11]. Prophylactic atropine should be administered in patients with these potential risk factors for hemodynamic instability during brachiocephalic artery stenting.

Limitations inherent to a case report are lack of ability to generalize, no possibility to establish a cause-effect relationship, danger of over-interpretation, publication bias, and retrospective design [12].

## Conclusions

Hypotension and bradycardia can occur after brachiocephalic artery stenting. Three presumptive mechanisms may explain the occurrence of hypotension and bradycardia in this case. First, improved blood flow in the carotid artery due to brachiocephalic artery stenting can stimulate carotid sinus baroreceptors. Second, baroreceptors in the aortic arch may be stimulated by the protrusion of the brachiocephalic artery stent into the aortic arch. Finally, baroreceptors potentially located in the brachiocephalic artery may contribute to this hemodynamic instability. More histological studies are needed to understand the anatomical innervation of the brachiocephalic artery. This hemodynamic instability should be noted when performing interventions for brachiocephalic artery lesions. Future studies are warranted to examine the frequency and risk factors of hypotension and bradycardia in brachiocephalic artery stenting and the effects of this hemodynamic instability on patient outcomes.
